# Dietary Omega-3 Fatty Acids Do Not Change Resistance of Rat Brain or Liver Mitochondria to Ca^2+^ and/or Prooxidants

**DOI:** 10.1155/2012/797105

**Published:** 2012-08-27

**Authors:** Irina G. Stavrovskaya, Susan S. Bird, Vasant R. Marur, Sergei V. Baranov, Heather K. Greenberg, Caryn L. Porter, Bruce S. Kristal

**Affiliations:** ^1^Department of Neurosurgery, Brigham and Women's Hospital, 221 Longwood Avenue, Room LM322, Boston, MA 02115, USA; ^2^Department of Surgery, Harvard Medical School, Boston, MA 02115, USA; ^3^Department of Neurological Surgery, Presbyterian Hospital, University of Pittsburgh Medical Center, Pittsburgh, PA 15213, USA

## Abstract

Omega-3 polyunsaturated fatty acids (n-3 PUFAs) block apoptotic neuronal cell death and are strongly neuroprotective in acute and chronic neurodegeneration. Theoretical considerations, *indirect* data, and consideration of parsimony lead to the hypothesis that modulation of mitochondrial pathway(s) underlies at least some of the neuroprotective effects of n-3 PUFAs. We therefore systematically tested this hypothesis on healthy male FBFN1 rats fed for four weeks with isocaloric, 10% fat-containing diets supplemented with 1, 3, or 10% fish oil (FO). High resolution mass spectrometric analysis confirmed expected diet-driven increases in docosahexaenoic acid (DHA, 22:6, n-3) and eicosapentaenoic acid (EPA, 20:5, n-3) in sera, liver and nonsynaptosomal brain mitochondria. We further evaluated the resistance of brain and liver mitochondria to Ca^2+^ overload and prooxidants. Under these conditions, neither mitochondrial resistance to Ca^2+^ overload and prooxidants nor mitochondrial physiology is altered by diet, despite the expected incorporation of DHA and EPA in mitochondrial membranes and plasma. Collectively, the data eliminate one of the previously proposed mechanism(s) that n-3 PUFA induced augmentation of mitochondrial resistance to the oxidant/calcium-driven dysfunction. These data furthermore allow us to define a specific series of follow-up experiments to test related hypotheses about the effect of n-3 PUFAs on brain mitochondria.

## 1. Introduction

In mammals, the central nervous system (CNS) has the second highest concentration of lipids after adipose tissue. Lipids play a critical role in neuronal membrane function as well as in enzyme, receptor, and ion channel activities [1, 2]. One of the main constituents of brain phospholipids is the omega-3 group of polyunsaturated fatty acids (n-3 PUFAs). There are three major n-3 PUFAs: alpha-linolenic (ALA), eicosapentaenoic (EPA), and docosahexaenoic (DHA) acids. DHA (22:6, n-3), the longest and most unsaturated fatty acid, is an essential n-3 PUFA for brain—it is highly enriched in neural membranes, constituting 30–40% of phospholipids in the cerebral cortex and retina [3, 4]. Because brain tissue is unable to make n-3 PUFAs, it is remarkably sensitive to adequate diet supplementation during all stages of CNS development—from embryonic differentiation to adulthood and aging [2, 4–7]. Neural trauma and neurodegeneration are associated with significant disturbances in neuronal membrane phospholipid metabolism [8–10], suggesting that supplementation with n-3 PUFAs may be beneficial for recovery.

Omega-3 deficiency induces structural and functional abnormalities in the hippocampus, hypothalamus, and cortex-brain areas that mediate spatial and serial learning [7]. Omega-3 deficiency significantly reduces the level of cerebral catecholamines, brain glucose transport capacity and glucose utilization, cyclic AMP level, and the capacity for phospholipid synthesis. Such a deficiency also markedly affects activity of membrane-bound enzymes, receptors and ion channels (e.g., Na^+^, K^+^-ATPase), production of neurotransmitters and brain peptides, gene expression, intensity of inflammation, and synaptic plasticity [1, 7, 11, 12]. Conversely, diet supplementation with DHA modulates gene expression, neurotransmitter release, restores synaptic activity reduced by age or trauma, and improves memory and learning abilities [10, 13–19], while the effect of n-3 PUFAs on membrane fluidity remains to be a controversial [20].

Numerous studies conducted over the past decade suggest that n-3 PUFA has a significant neuroprotective and proregenerative potential [21–30]. Particularly, acute intervention or dietary supplementation with n-3 PUFAs have been found to be protective in animal models of acute neurologic injury such as cerebral stroke, traumatic brain and spinal cord injuries [23–26, 28–30], and some case studies [21]. Recent study has demonstrated the improved outcome after peripheral nerve injury in transgenic mice with elevated level of endogenous n-3 PUFA [22].

The neuroprotective properties of n-3 PUFAs are in part attributed to their strong anti-inflammatory action, mediated partially by DHA's inhibition of AA catabolism and modulation of cytokine levels, and antioxidant potential [11, 12]. It has been recently demonstrated that after the onset of brain injury, DHA could be released from membrane phospholipids by Ca^2+^-dependent phospholipase A_2_ and generates neuroprotective D1—a compound that differentially regulates the expression of pro- and antiapoptotic proteins from Bcl-2 family, known as a critical players in cell fate [31]. Despite the wide range of targets and proposed mechanisms of n-3 PUFA beneficial action, the remaining question is how they (e.g., targets and mechanisms) are activated in order to execute these effects.

Within the cell, the mitochondrial membrane is one of the primary sites for n-3 PUFA incorporation along with endoplasmatic reticulum and plasma membrane [14, 32–35]. Brain, cardiac and liver mitochondria fatty acids turnover requires 3-4 weeks and is highly regulated by diet [34–36]. A growing body of evidence has established that mitochondria are a key component in the signaling pathway(s) underlying cell death [16, 36–41]. To some extent, mitochondria serve to integrate different apoptosis-inducing stimuli (Ca^2+^, proapoptotic Bcl-2 family proteins, reactive oxygen species, etc.) and direct them into a common downstream pathway [36, 37, 39, 41]. Mitochondria are enlisted to initiate the downstream stages of cell death through mitochondria-permeability-transition-(MPT) dependent and -independent mechanisms. The MPT is a multiprotein pore complex of as yet unidentified structure that is presumably localized at the contact sites between the inner and outer mitochondrial membranes. The MPT begins as a permeabilization of the inner membrane, which prevents buildup of a mitochondrial membrane potential and leads to loss of the ability to sequester calcium from the medium, progressive osmotic swelling, disruption of the outer membrane, loss of matrix and intermembrane proteins, and initiation of caspase-dependent and caspase-independent cell death pathways [36, 39]. Mitochondrial damage, occurring via the MPT, has been identified as a critical event in stroke and stroke-related injuries, secondary injury following brain trauma (TBI), and chronic neurodegeneration [16, 21, 23–27, 36, 38, 39, 41–43].

In light of the aforementioned links between mitochondria and cell death, mitochondria and n-3 PUFAs, and n-3 PUFAs and neuronal function, it is noteworthy that recent evidence shows that n-3 PUFAs can modulate processes that contribute to the secondary degeneration of the CNS [10, 17–19, 44, 45]. Particularly, administration of n-3 PUFAs after spinal cord compression injury in rats significantly increased neuronal survival and improved locomotive performance for up to 6 weeks after injury. Furthermore, preinjury diet supplementation with omega-3 PUFAs prevented some TBI-induced effects—a reduction in synaptic plasticity and impaired learning ability and reduced oxidative damage. Recent data suggest that eight weeks of dietary supplementation with DHA delays induction of MPT mediated swelling and increases ability to retain exogenously added calcium in cardiac mitochondria [46]. These data, coupled with the above background, suggests that diets enriched in n-3 PUFAs might affect mitochondria in a way that makes them more resistant to the oxidant- and calcium-mediated injury associated with both acute neurological injury and induction of the MPT.

The goal of present study was, therefore, to test directly the involvement of the MPT pathway in n-3 PUFA-mediated protection in brain and liver mitochondria isolated from healthy rodents. Specifically, we determined whether 4 weeks dietary supplementation in rats with 1, 3, or 10% FO, containing essential n-3 PUFAs—EPA and DHA, changes the resistance of isolated nonsynaptosomal brain and liver mitochondria to proapoptotic signals such as Ca^2+^ and prooxidants.

## 2. Material and Methods

### 2.1. Chemicals

 Tetramethylrhodamine methyl ester (TMRM) and Ca-Green-5N were purchased from Invitrogen, Inc. (Carlsbad, CA). All other chemicals were purchased from Sigma-Aldrich Company (St. Louis, MO). IsoKs were synthesized as described previously [47]. LC-MS grade acetonitrile (ACN), methanol (MeOH), and isopropanol (IPA), as well as high-performance liquid chromatography (HPLC) grade dichloromethane (DCM) and dimethyl sulfoxide were purchased from Fisher Scientific (Pittsburg, PA) and ammonium formate was purchased from Sigma-Aldrich (St. Louis, MO). Lipid standards purchased for LC-MS as well as their abbreviations and sources are in the supplemental information (Tables S1 and S2) from [48, 49].

### 2.2. Dietary Supplementation

This study was carried out in strict accordance with the recommendations of the guide for the Care and Use of Laboratory Animals of the National Institutes of Health. The protocol was approved by the Harvard Medical School Standing Committee on Animals (Protocol no. 04381).

Ninety-six male Fisher 344 x Brown Norway F_1_ (FBFN_1_) rats (four groups, 24 animals in each), three weeks of age were housed in cages (two rats per cage) and maintained in an environmentally controlled room. All animal procedures were performed in accordance with the Guide for the Care and Use of Laboratory Animals and were approved by the Animal Studies Committee at Harvard University. After acclimatization for 1 week on standard rat chow, the rats were randomly assigned to diets supplemented with fish oil (FO) containing n-3 PUFAs—DHA and EPA (final FO 1, 3, or 10% of total fat in a fixed 10% fat diet, i.e., the 10% FO diet is 1% omega-3 PUFAs by weight) for 4 weeks. In all diets, including in the control diet, the total fat content made up 10% of the diet by weight; of that fat component, total saturated fats made up 30%, total monounsaturated fats made up 26%, and total polyunsaturated fats made up 44% (Table S1). The diets, fed *ad libitum, *were provided as pellets (Research Diets, Inc.) and contained a standard vitamin and mineral mix with all essential nutrients. Diet also contained antioxidants to preserve FO from the oxidation. After 4 weeks on these diets, rats were decapitated. Brain and liver tissues were rapidly dissected and placed into ice-cold buffer for isolation of mitochondria.

Greater details of the husbandry and diets are presented in the supplementary material following the ARRIVE criteria for reporting animal studies [50].

### 2.3. Mitochondrial Isolation

Nonsynaptosomal brain mitochondria were isolated from ~2-3-month-old rats using a discontinuous Ficoll gradient according to the commonly used method of Lai and Clark [51], with slight modifications as previously described [52].

Liver mitochondria were isolated from ~2-3-month-old rats by the standard differential centrifugation method in sucrose-based buffers as described and as used previously in our lab [40]. Liver isolation buffer contained 0.3 M sucrose, 10 mM HEPES, 1 mM EGTA, and 0.5% bovine serum albumin (BSA). Mitochondrial protein concentration was determined by the Lowry method using BSA as a standard [53].

Mitochondrial yield was identical in all samples from all tested dietary groups for both tissues.

### 2.4. Plasma and Isolated Mitochondria Lipid Extraction, LC-MS Conditions, Data Analysis, and Lipid Identification

 Immediately before extraction, brain and liver mitochondria isolated from each animal had their membranes disrupted by sonication. Both a brain and a liver mitochondrial pool sample were created by combining 10 *μ*L from the sonicated mitochondria of each rat in addition to a serum pool. These samples were processed for quality control (QC) and lipid identification studies at the same time as the dietary samples.

Lipids were extracted according to the method of Bligh and Dyer [54], substituting DCM for chloroform [42]. First, 10 *μ*L of an internal standard mixture containing 5 lipids as outlined in detail previously [48, 49] was added to each 30 *μ*L sample (either mitochondria or serum), followed by 190 *μ*L of MeOH, 20 seconds of vortexing, 380 *μ*L of DCM, and 120 *μ*L of water was added to induce phase separation. The samples were then vortexed for 10 seconds and allowed to equilibrate at room temperature for 10 minutes before centrifugation at 8000 g for 10 minutes at 10°C. A total of 320 *μ*L of the lower lipid-rich DCM layer was then collected and the solvent evaporated to dryness under vacuum. Samples were reconstituted in 300 *μ*L of ACN/IPA/H_2_O (65 : 30 : 5 v/v/v) containing PG (17 : 0/17 : 0) at a concentration of 1 *μ*g/mL before LC-MS analysis. Ten *μ*L of sample was injected onto the LC-MS system.

Lipids were extracted from 30 *μ*L serum and mitochondria aliquots of all individual rat samples and total study pool samples, created by taking aliquots from each sample as described previously [48, 49]. Ten *μ*L of sample was injected onto the LC-MS system. Details of the LC-MS method have been described previously [48, 49]. Briefly, lipid extracts were separated on an Ascentis Express C_18_ 2.1 × 150 mm 2.7 *μ*m column (Sigma-Aldrich, St. Louis, MO) connected to a Thermo Fisher Scientific autosampler and Accela quaternary HPLC pump (Thermo Fisher, San Jose, CA). A binary solvent system was used, in which mobile phase A consisted of ACN : H_2_O (60 : 40), 10 mM ammonium formate, 0.1% formic acid and mobile phase B of IPA : ACN (90 : 10), 10 mM ammonium formate, 0.1% formic acid. Separations were done over a 30-minute-period following the conditions set by Hu and colleagues [55]. The HPLC system was coupled to an Exactive benchtop orbitrap mass spectrometer (Thermo Fisher, San Jose, CA) equipped with a heated electrospray ionization (HESI) probe. For full scan only experiments, the MS was operated between m/z 120–2000 in high resolution mode, corresponding to a resolution of 60 k and a 2 Hz scan speed. The instrument was tuned by direct infusion of PG (17 : 0/17 : 0) in both positive and negative mode, and external mass calibration was performed according to the manufacturer's protocol. HCD experiments were performed by alternating between full scan acquisitions and HCD scan acquisitions, both run at 2 Hz. Three different HCD energies, 30, 60, and 100 eV, were used in separate experiment as previously described. For lipid identification studies, HCD experiments were run on the pool samples only. Results from all LC-MS profiling experiments were analyzed using the MS label free differential analysis software package SIEVE v 1.3 (Thermo Fisher Scientific and Vast Scientific, Cambridge, MA) (for details see [48, 49]).

### 2.5. Mitochondrial Respiratory Assay

 Mitochondrial respiration was measured with the Oxygraph 2 k electrode system from Oroboros Instruments (http://www.oroboros.at). Brain mitochondria (0.25 mg/mL) were incubated in buffer containing 100 mM sucrose, 65 mM KCl, 10 mM HEPES, pH 7.4, 2 mM KH_2_PO_4_, 3 *μ*M EDTA, and 5 mM glutamate/malate. Liver mitochondria (0.25 mg/mL) were incubated in buffer containing 250 mM sucrose, 10 mM HEPES, pH 7.4, 1 mM KH_2_PO_4_, and 5 mM succinate + 1 *μ*M rotenone.

Isolated brain or liver mitochondria were added after the addition of 5 mM glutamate/malate (complex I substrate) or 5 mM succinate + rotenone (complex II substrate) into media. Respiration in the presence of substrates corresponds only to state 2 respiration (*V*_2_). Addition of 200 *μ*M ADP initiates ATP synthesis coupled to proton reentry across the membrane, which corresponds to state 3 (*V*_3_). ADP exhaustion leads to a reduction in the respiratory rate and corresponds to state 4 (*V*_4_). Addition of 0.5 *μ*M of the ionophore carbonyl cyanide-p-trifluoromethoxyphenylhydrazone (FCCP) induced uncoupled respiration (*V*_unc_). The respiratory control ratio (RCR) was calculated as the ratio between the rates of respiration in states 3 and 2 (by Lardy, RCR_3/2_); states 3 and 4 (by Chance, RCR_3/4_); FCCP-stimulated respiration and state 3 (*V*_unc_/*V*_ADP_). 

### 2.6. Measurement of Mitochondrial Ca^2+^ Uptake Capacity, Membrane Potential, and NAD(P)H/NADH Oxidation and Swelling

The measurement of these parameters was performed simultaneously on a multichannel dye fluorimeter (C&L Instruments, Inc., http://www.fluorescence.com/). Liver mitochondria were incubated in buffer containing 250 mM sucrose, 10 mM HEPES, pH 7.4, 1 mM KH_2_PO_4_, and 5 mM glutamate/malate or succinate and were used at a concentration of 0.25 mg/mL mitochondria [43, 52]. Brain mitochondria were incubated in buffer containing 100 mM sucrose, 65 mM KCl, 10 mM HEPES, pH 7.4, 2 mM KH_2_PO_4_, 150 *μ*M ATP, 150 *μ*M MgCl_2_, 3 *μ*M EDTA, and 5 mM glutamate/malate at a concentration of 0.10 mg/mL mitochondria [52].

The mitochondrial membrane potential changes (ΔΨ) were estimated by measuring changes in the fluorescence intensity of TMRM (60 nM) (molecular probes) at excitation and emission wavelengths of 543 and 590 nm, respectively.

Mitochondrial Ca^2+^ fluxes were measured as changes of extramitochondrial [Ca^2+^], which were followed by monitoring the fluorescence intensity of Ca-Green-5N (125 nM) (Invitrogen) at excitation and emission wavelengths of 482 and 535 nm, respectively. Mitochondria were challenged to single or multiple Ca^2+^ additions. For liver mitochondria, each addition was 20–40 nmol Ca^2+^/mg mitochondrial protein; for brain mitochondria, each addition was 200 nmol/mg protein. Mitochondrial calcium retention capacity (CRC) was determined as amount of Ca^2+^ sequestered by mitochondria without incurring structural damage and expressed in nmol/mg protein. The redox state of pyridine nucleotides in the mitochondrial suspension was followed by monitoring NADH autofluorescence at excitation and emission wavelengths of 350 and 450 nm, respectively.

Mitochondrial swelling was measured as a function of light scattering at excitation and emission wavelengths of 587 nm or by a standard spectroscopic assay on a plate reader at 540 nm.

Original respiration and fluorimeter-based data were analyzed using Origin v.8.0 (OriginLab) software. Data were normalized to protein concentration and expressed in corresponding units.

### 2.7. Kinetic Analysis of the Ca^2+^-Induced MPT in Liver Mitochondria

**Scheme 1:**
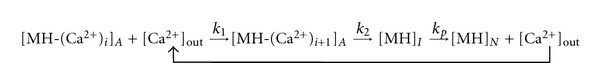


We used a recently developed kinetic model of Ca^2+^-induced MPT [56] to analyze the effects of the supplemented diets on liver mitochondrial function. The model describes MPT induction as a series of 2 sequential steps defined as Ca^2+^-uptake (*k*_1_) and formation of an intermediate step (*k*_2_) followed by mitochondrial membrane permeabilization accompanied with a putative MPT pore opening (*k*_*p*_), Scheme 1. In the framework of the model at least 3 possible mechanisms of action for different modulators of mitochondrial dysfunction could be resolved. Numerical analysis of the kinetics of simultaneously measured Ca^2+^ fluxes and swelling provides information on the contribution of each of the steps into the process of MPT induction.

The rate *k*_1_ is easily assigned to the Ca^2+^ uptake by active mitochondria [MH-(Ca^2+^)_i_]_A_ via Ca^2+^ uniporter. The formation of an intermediate state [MH]_I_, on the other hand, is a complex function of the number of Ca^2+^ ions absorbed by the mitochondrion and expressed as *k*_2_ = *k*__2__′ × ([Ca^2+^]_*M*_/[MH]_*A*_)^*n*^. Here, [Ca^2+^]_M_ is the concentration of Ca^2+^ that has been absorbed by mitochondria; thus, the ratio [Ca^2+^]_M_/[MH]_A_ is essentially an average number of Ca^2+^ ions absorbed per active mitochondrion. Parameter *n* is an apparent order of the *k*_2_ step with respect to Ca^2+^, and *k*__2__′ is the reaction constant of this step. Kinetic parameter *k*_2_ is related to the ability of the Ca^2+^ sequestered by mitochondria to facilitate the formation/assembly of the MPT pore intermediate. Functionally, the intermediate represents MPT pore assembled but still closed. In our model, parameter *n* interpreted as an order of reaction reflects quantitative characteristics of MPT intermediate formation in respect to Ca^2+^. Therefore, any change in *n* would be expected to correlate with an ability of mitochondria to resist Ca^2+^-induced MPT.

### 2.8. Statistical Analysis

Data were presented as the mean +/− the standard error of the median (SEM). Group comparisons of the effects of diet modulation on mitochondrial parameters were determined by a two-sample *t*-test (Origin 8.0). The significance of data was considered at *P* value 0.05. Experiments were performed 5-6 times.

## 3. Results

Rats consumed 14.1 ± 3.1 g of food a day and gained 4.85 ± 1.55 g of body weight a day for 28 days. Food intake and body weight gain of all the groups were not significantly different.

### 3.1. Plasma and Mitochondria Fatty Acid Composition

**Figure 1:**
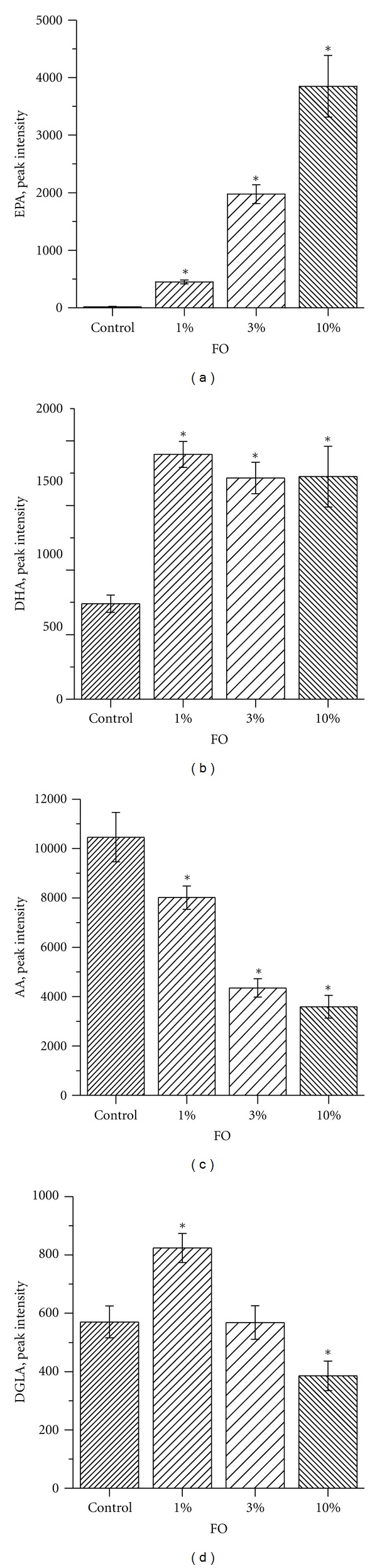
Effect of dietary FO on plasma level of EPA, DHA, AA, and DGLA. Data are expressed as means of group of *n* = 6, ±SEM. Statistical significance between tested samples was determined by a two-sample *t*-test, level of significance was set at *P* < 0.05.

**Figure 2:**
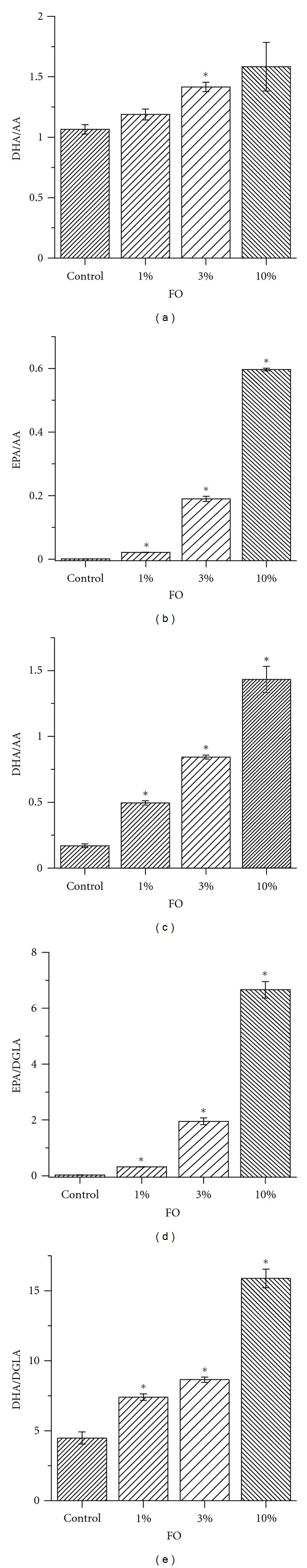
Effect of dietary FO on mitochondrial ratio of EPA, DHA, AA, and DGLA. (a) Changes in nonsynaptosomal brain mitochondria fatty acids; (b–e) Changes in liver mitochondria. Data are expressed as means of group of *n* = 3–5, ±SEM. Statistical significance between tested samples was determined by a two-sample *t*-test, level of significance was set at *P* < 0.05.

By using LC-MS profiling, we were able to monitor the n-3 and n-6 free fatty acids—EPA, DHA, AA and the AA, synthesis intermediate DGLA (20:3, n-6) in both the serum and mitochondrial samples. This method did not provide absolute quantitative amounts of the fatty acids in each sample; however, it provided a means to quantitatively monitor these species across the biological samples. The study gives information regarding relative amounts of species present and allows for direct comparisons across samples to be performed [48, 49]. The FO diets increased the level of the free fatty acid EPA in serum of tested animals in a dose-dependent manner (note that in a control diet it was almost absent) (Figure 1(a)). The sera level of DHA, in contrast, reached a plateau in the 1% FO dietary group (Figure 1(b)). Conversely, the level of n-6 fatty acids—AA and DGLA were both reduced with an increase in n-3 PUFAs supplementation. The dose dependency of this reduction was more pronounced for AA than for DGLA. Diet-driven fatty acid changes in mitochondria (Figure 2) were similar to those seen in sera. These changes were monitored by comparing the ratios of the n-3 to n-6 species. The LC-MS profiling method assumes uniform starting material concentration. For lipid extraction, the mitochondria were not uniformly concentrated based on amount of protein; therefore, the signal was standardized by comparing the ratio of n-3 to n-6 which should be consistent regardless of starting amounts. The ratio of DHA to AA in brain mitochondria was particularly elevated with a maximum in 3% FO dietary group (Figure 2(a)). Brain mitochondria level of EPA was very low and dietary-driven changes were undetectable. FO dietary supplementation induced a dose-dependent elevation of the ratios of EPA to AA, DHA to AA, EPA to DGLA, and DHA to DGLA (Figures 2(b)–2(e)).

### 3.2. Brain and Liver Mitochondria Respiratory Parameters

Respiratory parameters of brain and liver mitochondria isolated from tested animal groups were measured in different metabolic states: (state 2 (*V*_2_), state 3 (*V*_3_), state 4 (*V*_4_), FCCP-stimulated, or uncoupled respiration (*V*_unc_)). The substrate glutamate/malate was used to stimulate respiration initiated at complex I and the substrate succinate + rotenone was used to stimulate respiration initiated at complex II.

**Figure 3:**
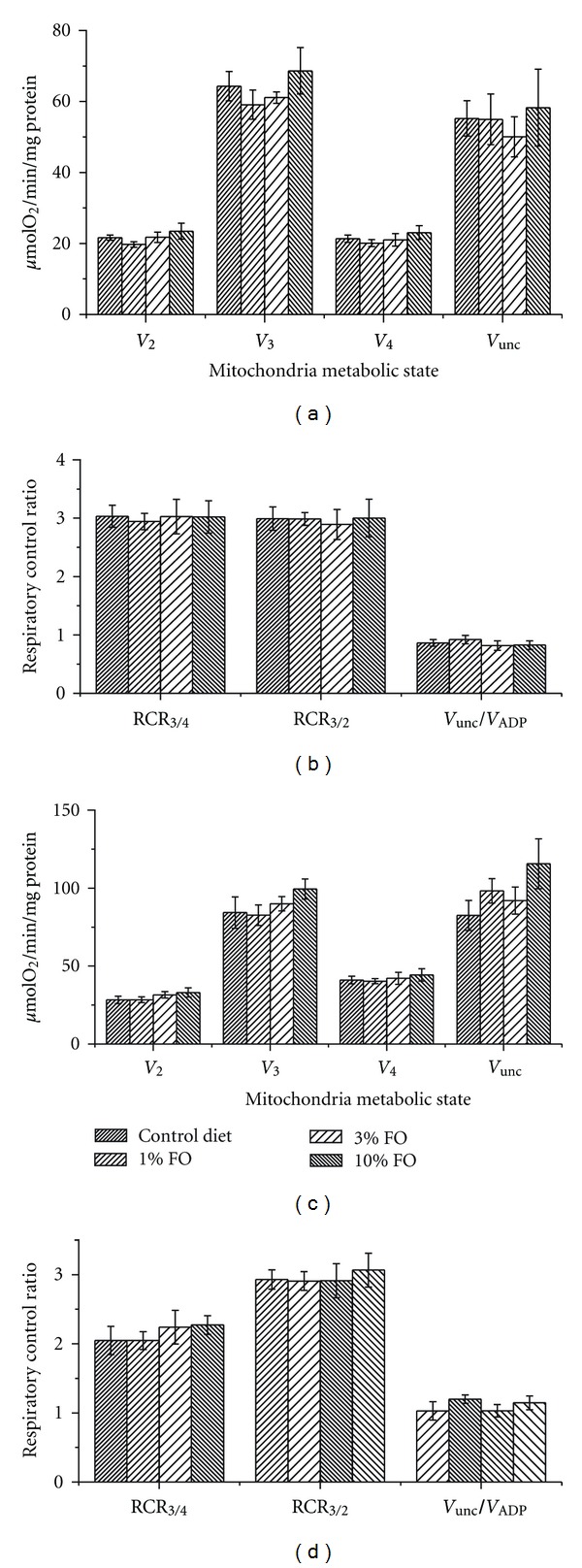
Omega-3 PUFA enriched diet does not affect brain mitochondria respiratory parameters. (a and c) Respiration rates measured in different metabolic states state 2 (*V*_2_), state 3 (*V*_3_), state 4 (*V*_4_), FCCP-stimulated respiration (*V*_unc_). (b and d) RCRs. In (a and b), glutamate/malate was used to stimulate respiration initiated at complex I; in (c and d), succinate and rotenone were used to stimulate respiration initiated at complex II. The data are expressed in *μ*mol O_2_/min/mg protein and are the mean ± SEM, *n* = 6. Statistical significance in oxygen consumption between tested samples was determined by a two-sample *t*-test.

Brain mitochondria isolated from rats fed FO diet had higher rates of ADP- and FCCP-stimulated respiration (*V*_ADP_ and *V*_unc_, resp.), with succinate as a substrate (Figure 3(c)), but not with glutamate as a substrate (Figure 3(a)). RCR_3/2_, RCR_3/4_, and RCR_*V*_unc_/*V*_ADP__, calculated as the ratios of respiration rates between state 3/state 2, state 3/state 4, and uncoupled state/state 3, respectively, were also unchanged by FO diet (Figures 3(b) and 3(d)).

**Figure 4:**
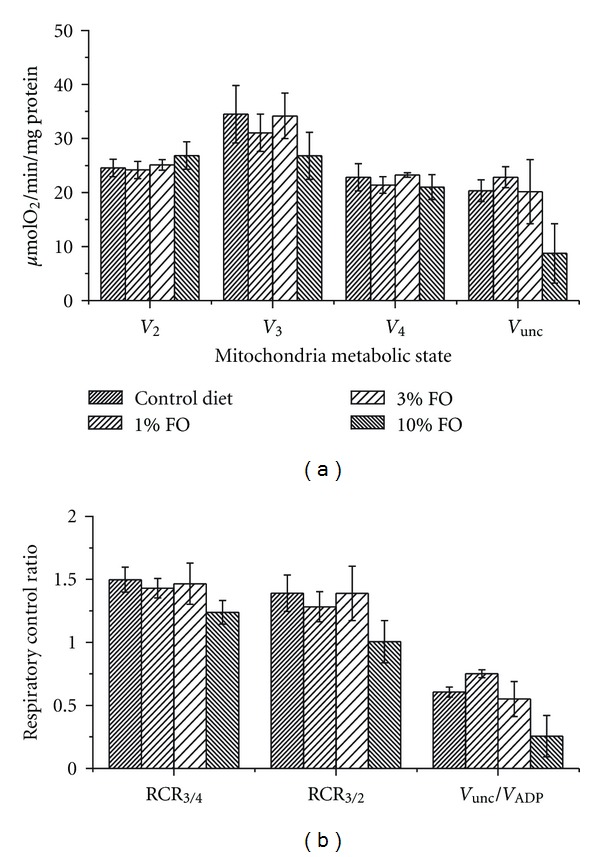
Omega-3 PUFA enriched diet does not affect the sensitivity of brain mitochondria respiratory parameters to IsoKs. (a) Respiratory parameters measured in different metabolic states state 2 (*V*_2_), state 3 (*V*_3_), state 4 (*V*_4_), FCCP-stimulated respiration (*V*_unc_). Glutamate/malate was used as the substrate. IsoKs were added to mitochondria after the addition of substrate. (b) The dependence of the RCRs on omega-3 dietary content. The data are expressed in *μ*mol O_2_/min/mg protein and are the mean ± SEM, *n* = 6. Statistical significance in oxygen consumption between tested samples was determined by a two-sample *t*-test.

Challenging brain mitochondria with isoketals (IsoKs, 2 *μ*M), which are prooxidative ketoaldehydes [47, 52], caused no statistically significant differences in the rates of respiration between mitochondria from the different dietary groups (Figure 4(a)). However, brain mitochondria isolated from animals fed 10% FO diet had lower rate of FCCP-stimulated respiration compared to mitochondria isolated from brain tissue of another dietary group. The RCR_3/4_, RCR_3/2_, and V_unc/ADP_ also were lower in the presence of IsoKs in all tested animal groups, but this observation was similar for all diets (Figure 4(b)).

**Figure 5:**
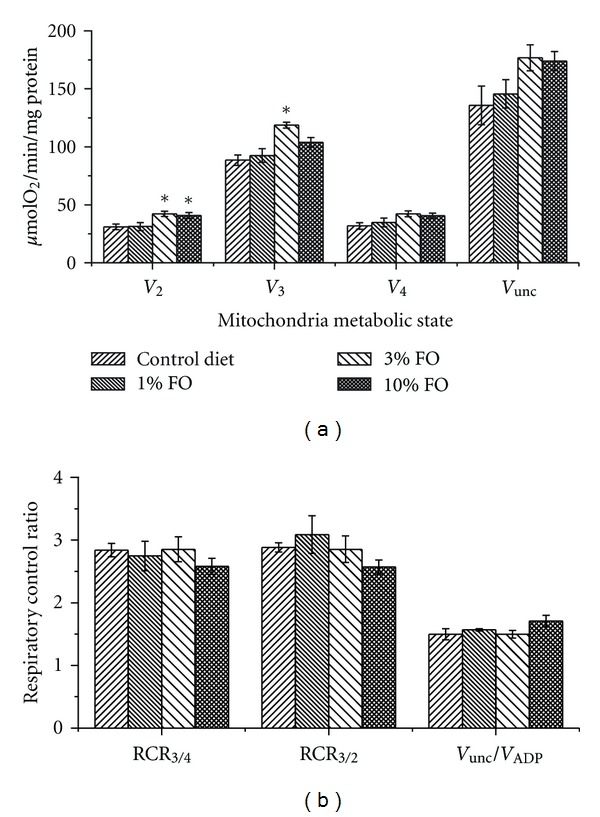
Omega-3 PUFA enriched diet does not affect liver mitochondria respiratory parameters. Respiratory parameters were measured in different metabolic states state 2 (*V*_2_), state 3 (*V*_3_), state 4 (*V*_4_), FCCP-stimulated respiration (*V*_unc_). (a) 5 mM succinate +1 *μ*M rotenone (1 *μ*M) was used to stimulate respiration initiated at complex II; (b) The effect of diets on RCRs measured on succinate + rotenone. The data are expressed in *μ*mol O_2_/min/mg protein and are the mean ± SEM, *n* = 6. Statistical significance in oxygen consumption between tested samples was determined by a two-sample *t*-test.

Respiratory parameters of liver mitochondria were measured similarly to those of brain mitochondria (Figure 5). As shown, FO 1–10% dietary supplementation did not induce statistically significant changes in liver mitochondria respiration.

### 3.3. Nonsynaptosomal Brain Mitochondria Δ*μ*, Ca^2+^ Uptake/Release, and Redox State of Pyridine Nucleotides and Swelling

**Figure 6:**
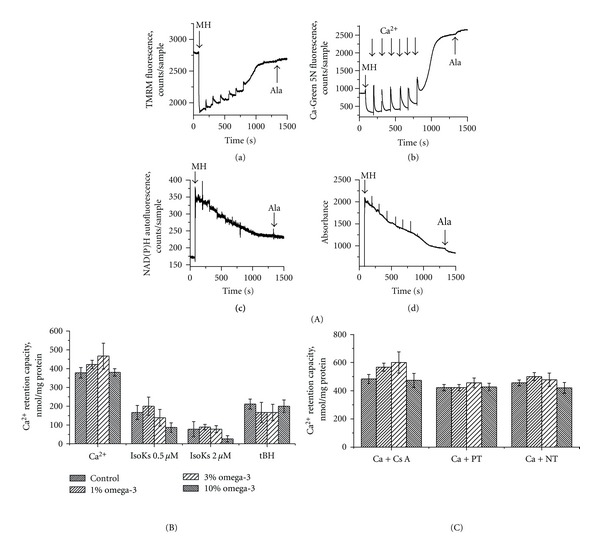
Omega-3 PUFA enriched diet does not change brain mitochondria Ca^2+^ retention capacity. (A) Basic parameters of isolated from control rats brain mitochondria measured in the presence of the chain of Ca^2+^ additions (10 *μ*M each addition) and 5 mM glutamate/malate as a substrate. (a) Mitochondrial ΔΨ was measured as changes in TMRM fluorescence signal. Addition of mitochondria into incubation buffer, indicated by arrow, induced the drop of TMRM signal (i.e., TMRM concentration in the buffer) due to the accumulation of the dye by mitochondria. The addition of Ca^2+^ induced an increase of TMRM signal, which corresponds to lower ΔΨ, and a subsequent decrease of the TMRM signal, which indicates a restoration of ΔΨ. (b) Ca^2+^ concentration in the mitochondrial suspension was measured as Ca-Green 5N fluorescence signal. A higher signal corresponds to an increase of Ca^2+^ concentration in the buffer. (c) NAD(P)H autofluorescence signal. A lower signal corresponds to a more oxidized state of NAD(P)H. (d) Mitochondrial swelling was measured as changes in light scattering of the mitochondrial suspension. A decrease in the fluorescence signal indicates an increase in swelling. Spikes resulting from additions of Ca^2+^ were manually reduced; arrows are used to indicate where these manipulations occurred. Representative traces from 6 experiments are shown. (B) CRC determined in the presence of Ca^2+^, Ca^2+^ + IsoK (0.5 and 2 *μ*M), Ca^2+^ + tBH (100 *μ*M); (C) Ca^2+^ + Cs A (0.5 *μ*M), Ca^2+^ + promethazine (PT) (3 *μ*M), and Ca^2+^ + nortriptyline (NT) (3 *μ*M), use (B) for comparison. Glutamate/malate (5 mM) was used as the substrate. The data are expressed in nmol of Ca^2+^ consumed by mitochondria per mg of mitochondrial protein and are the mean ± SEM, *n* = 6. Statistical significance in CRC between tested samples was determined by a two-sample *t*-test.

Figure 6(a) shows a representative record of basic parameters (e.g., Δ*μ*, Ca^2+^ uptake/release, redox state of pyridine nucleotides and swelling) of nonsynaptosomal brain mitochondria isolated from control group of animals. Further assessment of brain mitochondria function suggested no statistically significant difference between tested groups. However, mitochondria isolated from animals fed FO 1% or 3% diets showed a weak tendency to be more resistant to a Ca^2+^ challenge (Figure 6(b)). Resistance was defined as an increase in Ca^2+^ retention capacity (CRC), that is, the ability to sequester Ca^2+^ without incurring structural damage. Cyclosporine (Cs A), the prototypical MPT pore inhibitor, increased the CRC in all tested groups, with potentially enhanced protection in the groups fed FO 1% and 3% diets (Figure 6(c)). Promethazine (PT) and nortriptyline (NT)—drugs that possess potent MPT inhibitory properties [40]—confer less protection to brain mitochondria than Cs A, but similar to all tested dietary groups (Figure 6(c)). Incubation of mitochondria with a low concentration of prooxidants (0.5 *μ*M IsoKs) resulted in a significant reduction of the CRC in all tested groups (Figure 6(b)), as we have shown previously [52]. Animals in the FO 10% group demonstrated a tendency to be more susceptible to IsoKs. IsoKs (2 *μ*M) also significantly reduced the CRC in all groups; the effect was again more pronounced in mitochondria isolated from 10% FO group. Mitochondria showed no diet-dependent difference in susceptibility to the prooxidative agent tBH.

### 3.4. Kinetic Parameters of Ca^2+^-Induced MPT in Liver Mitochondria

**Figure 7:**
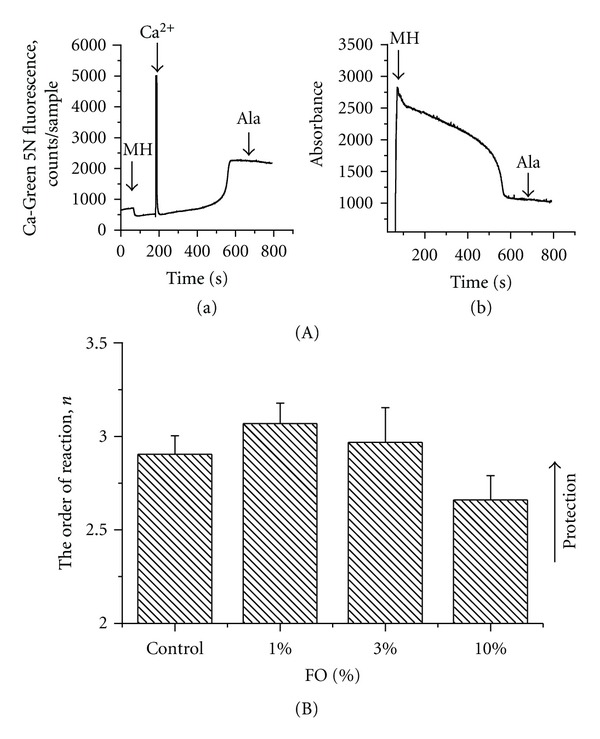
Dependence of kinetic parameter *n* (order of reaction) on dietary omega-3 PUFA content. (A) An ability of isolated from control animals liver mitochondria to uptake/release Ca^2+^ (section (a)) and swell (section (b)) in the presence of Ca^2+^ (10 *μ*M), succinate + rotenone used as a substrate. (B) The order of reaction *n* was calculated from our kinetic model of Ca^2+^-induced mitochondrial dysfunction for each tested diet. In the framework of the model (see description in text), a change in *n* reflects a change in the number of Ca^2+^ in respect to the MPT induction. Therefore, any change in *n* would be expected to correlate with an ability of mitochondria to resist to the Ca^2+^-induced swelling. The data are expressed as mean ± SEM, *n* = 3. Statistical significance of the differences in *n* between tested samples was determined by a two-sample *t*-test.

The kinetic analysis of the Ca^2+^ induced MPT (Figure 7) revealed that in all tested groups of animals, net Ca^2+^-uptake rate was not changed; that is, dietary intake did not affect Ca^2+^-uniporter activity nor modulated the factors that affect electrogenic Ca^2+^ uptake, such as ΔΨ, activity of *P*_*i*_ carrier, and activity of Ca^2+^ release pathways other than MPT (data not shown). The analysis of the rate of the formation of an MPT intermediate (*k*_2_-step) revealed that FO diets had no statistically significant changes in the resistance of mitochondria to Ca^2+^ (e.g., parameter *n*) (Figure 7), demonstrating only the tendency to be more protective (e.g., 1% or 3% FO diets) or harmful (e.g., 10% FO diet). 

## 4. Discussion

The results from our pilot study demonstrate that four weeks of dietary supplementation with 1, 3, or 10% FO enriched with an essential n-3 fatty acids EPA and DHA induced the expected significant changes in plasma and mitochondrial membrane fatty acid composition. The observed changes in brain and liver mitochondrial membrane phospholipid composition did not, however, significantly influence their resistance to Ca^2+^ and prooxidative agents (tBH and IsoKs). Only slight trend towards protective effects, insufficient to underlie the reported neuroprotective effects of n-3 PUFA supplementation, were ever seen with lower levels of supplementation (e.g., Figure 6(b)), and, higher levels of n-3 PUFAs supplementation were, if anything, detrimental. While these trends could be more systematically studied with very large experimental protocols, this would not advance our understanding of protection, and many spurious and nonrobust statistical observations would be mathematically expected to result from multiple comparison-related trends. Therefore, we do not believe that higher *N* studies are scientifically warranted.

Detailed evaluation of brain and liver mitochondrial function demonstrated almost no diet-associated changes in respiration, membrane potential, Ca^2+^ transport, redox state of pyridine nucleotides, or swelling, despite the elevation of EPA and DHA and the reduction of AA content in mitochondrial membrane. We also did not find differences in the sensitivity of mitochondria to the recently discovered MPT inhibitors promethazine and nortriptyline [40, 41] or the prototypical MPT inhibitor Cs A. The most parsimonious explanation is that n-3 PUFAs do not exert neuroprotection by augmenting mitochondrial resistance to calcium and oxidants. This explanation contradicts the hypothesis we initially proposed.

### 4.1. Reconsidering the Data: What Possibilities Remain?

The data presented contradict our proposed hypothesis, suggesting another explanation must link dietary omega-3 fatty acids with the inhibition of cell death cascades that involve mitochondria. One possibility is that other mitochondrial pathways (e.g., involving Bcl-2 family members—bcl-2, bax, bid, bak, etc.) are involved. Although this explanation is feasible, the injury used in experiments discussed earlier [17, 44, 45] is most consistent with the involvement of oxidant- and calcium-mediated damage, rendering the possibility of other mitochondrial pathways being involved somewhat unlikely. Alternatively, differences in the dietary protocols used in different experiments/laboratories as well as the animals' health status (e.g., the presence of pathology or injury) might underlie the different results. A review of the literature revealed at least two critical parameters that might influence the outcome: (i) the dosage of n-3 fatty acids dietary supplementation and (ii) the total fat content in the diet. These parameters vary substantially across the studies. The dosage of FO used in dietary studies varies up to 60% of total fats. The suggested n-3 PUFA therapeutic dose for humans is 1 to 3 g/day, which is equivalent to 1% FO diet for animals in the present study. The 10% FO diet in our study corresponds to an abnormally high dose for humans (approx. 30 g of n-3 PUFA/day), which is never used. Our data suggest that this dosage does not induce dramatic beneficial changes in mitochondrial functions; in fact, it tended to worsen them (e.g., it caused brain mitochondria to become more sensitive to Ca^2+^ and prooxidants and accelerated some respiratory parameters in liver). The lowest dose used in the present study, that is, the 1% FO diet that corresponds to an acceptable intake in humans showed no effect on the mitochondrial parameters tested. Recent study [43] suggests that a supplementation of healthy humans with DHA has dose-dependent effect: 200–400 mg/day of DHA had antioxidant effect on low-density lipoproteins, whereas 800–1600 mg/day of DHA was associated with elevated level of reactive aldehydes (e.g., 4-hydrohyhexenal) in plasma. These data are broadly consistent with our own and previous evidence showing aldehyde-induced mitochondrial dysfunction [41, 52].

With respect to total dietary fat content, we noted that brain and liver mitochondria isolated from control animals in this study exhibited less sensitivity to Ca^2+^ than we usually observed in our routine experiments with identical rats fed diets that have a lower percentage of total fat (4.5% versus 10% in the present study). Specifically, CRC for brain mitochondria isolated from animals fed with 4.5% is equal to 250–300 nmol of Ca^2+^/mg protein, whereas CRC for tested in the present study animal groups vary from 360 to 460 nmol of Ca^2+^/mg protein. Kinetic analysis done on the Ca^2+^ induced MPT in liver mitochondria isolated from animals fed with 4.5% of fat revealed Ca^2+^ requirement (kinetic parameter *n*) to be about 2.27 ± 0.05 [56], while the present study demonstrated considerably elevated *n* (up to 3.1). Such an increase was found upon application of promethazine [56] or in the medium depleted of inorganic phosphate (unpublished data). We demonstrated previously [56] that well-characterized MPT protecting agents such as Mg^2+^ or promethazine increased *n*, thereby increasing the resistance of mitochondria to Ca^2+^-induced MPT. On the other hand, addition of very low (0.5 nM) concentration of FCCP decreased *n* (not shown) facilitating MPT induction via a decrease of the ΔΨ, which is in line with data published earlier [36]. These data are consistent with an argument that an increase in dietary percent fat is associated with increased resistance of isolated mitochondria to calcium-overload injury. Unfortunately, many of the critical papers in this area fail to report the percentage of total dietary fat, and/or their control and experimental FO-enriched diets have different total fat contents. Also, in some studies, the diet enrichment with n-3 PUFAs is conducted in such a way as to increase total fat content. These observations suggest the need to investigate the effects of total dietary fat content, as well as the effects of prolonged n-3 PUFAs supplementation, on mitochondrial function.

In conclusion, we tested and rejected one of the possible mechanisms linking n-3 PUFA dietary supplementation and brain mitochondria functional parameters and injury mechanisms. The absence of a protective effect of diet in an *in vitro *model of ischemic injury (Ca^2+^overloading and prooxidant exposure), despite the observed changes in plasma and mitochondrial fatty acid content, suggests that increased resistance to Ca^2+^- and oxidant-mediated mitochondrial damage is not central to the well-documented neuroprotection induced byn-3 PUFAs. Investigation of other potential mechanisms, such as extramitochondrial targets and a potential role of total fat, will require further study.

## Supplementary Material

The tables S1 and S2 belong to the supplementation in references 48,49. The supplementation of the present paper has Table S1 and ARRIVE as mentioned in “Materials and Methods” section 2.2. Pointed online address did not work.

## Abbreviations

PUFAs: Polyunsaturated fatty acids
FO: Fish oil
MPT: Mitochondrial permeability transition
tBH: Tert-butylhydroperoxide
IsoKs: *γ*-Ketoaldehydes isoketals
TBI: Traumatic brain injury
ALA: Alpha-linolenic acid
EPA: Eicosapentaenoic acid
DHA: Docosahexaenoic acid
AA: Arachidonic acid
DGLA: Dihomo-gamma-linolenic acid
RCR: Respiratory control ratio
CRC: Calcium retention capacity of mitochondria
TMRM: Tetramethylrhodaminemethyl ester
Cs A: Cyclosporine A
FCCP: Carbonyl cyanide-p-trifluoromethoxyphenylhydrazone.
