# Supplementary material for: Dietary Omega-3 Fatty Acids Do Not Change Resistance of Rat Brain or Liver Mitochondria to Ca2+ and/or Prooxidants
Source: J Lipids. 2012 Aug 27;2012:797105. doi: 10.1155/2012/797105 (PMC3434410; doi:10.1155/2012/797105)
Supplement: Supplementary file 1 — The tables S1 and S2 belong to the supplementation in references 48,49. The supplementation of the present paper has Table S1 and ARRIVE as mentioned in “Materials and Methods” section 2.2. Pointed online address did not work. [file 797105.f1.pdf]

Supplemental Table

	Control		1% Menhaden		3% Menhaden		10% Menhaden	
	gm%	kcal%	gm%	kcal%	gm%	kcal%	gm%	kcal%
Protein	22	21	22	21	22	21	22	21
Carbohydrate	59	57	59	57	59	57	59	57
Fat	10	22	10	22	10	22	10	22
Total		100		100		100		100
kcal/gm	4.1		4.1		4.1		4.1	
Ingredient	gm	kcal	gm	kcal	gm	kcal	gm	kcal
Casein, 80 mesh	200	800	200	800	200	800	200	800
DL-Methionine	3	12	3	12	3	12	3	12
Corn Starch	100	400	100	400	100	400	100	400
Maltodextrin	50	200	50	200	50	200	50	200
Sucrose	396.5	1586	396.5	1586	396.5	1586	396.5	1586
Cellulose, BW200	50	0	50	0	50	0	50	0
Coconut Oil, 101	23	207	20	180	15	135	0	0
Safflower Oil	47.5	428	44	396	33	297	0	0
Safflower Oil, High Oleic	24	216	21.4	193	16	144	0	0
Menhaden Oil	0	0	10.6	95	32	288	96	864
Linseed	1.5	14	0	0	0	0	0	0
Mineral Mix S10001	35	0	35	0	35	0	35	0
Vitamin Mix V10001	10	40	10	40	10	40	10	40
Choline Bitartrate	2	0	2	0	2	0	2	0
Total	942.5	3902	942.5	3902	942.5	3902	942.5	3902

C2, Acetic	0.0	0.0	0.0	0.0
C4, Butyric	0.0	0.0	0.0	0.0
C6, Caproic	0.1	0.1	0.1	0.0
C8, Caprylic	1.8	1.5	1.2	0.0
C10, Capric	1.4	1.2	0.9	0.0
C12, Lauric	10.9	9.5	7.2	0.1
C14, Myristic	4.2	4.4	5.0	6.8
C14:1, Myristoleic	0.0	0.0	0.0	0.0
C15:0 Pentadecanoic	0.0	0.1	0.2	0.5
C16, Palmitic	6.4	7.4	9.5	15.9
C16:1, Palmitoleic	0.0	1.0	3.0	9.1
C16:2, Hexadecanoic	0.0	0.0	0.0	0.0
C16:3 Hexadecatrianenoic	0.0	0.0	0.0	0.0
C16:4 Hexcadecatetraenoi	0.0	0.0	0.0	0.0
C17:0	0.0	0.1	0.2	0.5
C17:1	0.0	0.0	0.1	0.2
C18, Stearic	4.0	3.8	3.6	3.1
C18:1, Oleic	24.9	23.5	20.6	12.1
C18:2, Linoleic	40.9	37.9	29.1	2.8
C18:3, Linolenic	0.9	0.2	0.5	1.3
C18:4	0.0	0.3	1.0	3.0
C20, Arachidic	0.1	0.1	0.1	0.2
C20:1,	0.1	0.2	0.6	1.6
C20:2, Auricolic	0.0	0.0	0.1	0.3
C20:3 Bishompinolenic	0.0	0.0	0.1	0.4
C20:4, Arachidonic	0.0	0.2	0.6	1.7
C20:5, Eicosapentaenoic	0.0	1.6	4.9	14.8
C21:5, Heneicosapentaeno	0.0	0.1	0.2	0.7
C22, Behenic	0.0	0.0	0.0	0.1
C22:1, Erucic	0.0	0.0	0.1	0.2
C22:2	0.00	0.15	0.46	1.39
C22:4, Clupanodonic	0.0	0.0	0.0	0.1
C22:5	0.0	0.3	0.9	2.7
C22:6, Docosahexaenoic	0.0	1.2	3.5	10.4
C24, Lignoceric	0.0	0.0	0.0	0.1
C24:1	0.0	0.0	0.1	0.3
Total	95.6	95.0	93.9	90.5
Saturated (g)	28.9	28.2	27.9	26.8
Monounsaturated (g)	24.9	24.8	24.4	23.5
Polyunsaturated (g)	41.7	41.8	40.9	38.3
Saturated (%)	30	30	30	30
Monounsaturated (%)	26	26	26	26
Polyunsaturated (%)	44	44	44	42
n-6:n-3 ratio	45.8	11.3	3.1	0.3

ARRIVE Criteria

TITLE

1 Provide as accurate and concise a description of the content of the article as possible.

Dietary omega-3 fatty acids do not change resistance of rat brain or liver mitochondria to Ca^{2+} and/or pro-oxidants

ABSTRACT

2 Provide an accurate summary of the background, research objectives, including details of the species or strain of animal used, key methods, principal findings and conclusions of the study.

Omega-3 polyunsaturated fatty acids (n-3 PUFAs) block apoptotic neuronal cell death and are strongly neuroprotective in acute and chronic neurodegeneration. Theoretical considerations, *indirect* data, and consideration of parsimony lead to the hypothesis that modulation of the mitochondrial pathway(s) underlies at least some of the neuroprotective effects of n-3 PUFAs. We therefore systematically tested this hypothesis on male FBFN1 rats fed for four weeks with isocaloric, 10 % fat-containing diets supplemented with 1, 3 or 10 % fish oil. High resolution mass spectrometric analysis confirmed the expected diet-driven increases in docosahexaenoic acid (DHA, 22:6n-3) and eicosapentaenoic acid (EPA, 20:5n-3) in sera as well as in liver and nonsynaptosomal brain mitochondria. We further evaluated the resistance of brain and liver mitochondria to Ca^{2+} overload and pro-oxidants – a model for induction of the mitochondrial permeability transition (MPT), an event linked to cell death during ischemic stroke and traumatic brain injury. Under these conditions, neither mitochondrial resistance to Ca^{2+} overload and pro-oxidants, nor mitochondrial respiratory physiology (oxygen consumption, pyridine nucleotide redox status, membrane potential) are altered by n-3 PUFA diet, despite the expected incorporation of DHA and EPA in mitochondrial membranes and plasma. These data argue against the aforementioned hypothesis that augmentation of mitochondrial resistance to the oxidant/calcium-driven MPT underlies n-3 PUFA-mediated neuroprotection reported in the literature. These data furthermore allow us to define a specific series of follow-up experiments to test related hypotheses about the effect of n-3 PUFAs on brain mitochondria and neuroprotection.

INTRODUCTION: Background

3 a. Include sufficient scientific background (including relevant references to previous work) to understand the motivation and context for the study, and explain the experimental approach and rationale.

In mammals, the central nervous system (CNS) has the second highest concentration of lipids after adipose tissue. Lipids play a critical role in neuronal membrane function, as well as in enzyme, receptor and ion channel activities [1,6]. One of the main constituents of brain phospholipids is the omega-3 group of polyunsaturated fatty acids (n-3 PUFAs). There are three major n-3 PUFAs: alpha-linolenic (ALA), eicosapentaenoic (EPA) and docosahexaenoic (DHA) acids. DHA is an essential n-3 PUFA for brain. It is highly enriched in neural membranes, constituting 30-40% of phospholipids in the cerebral cortex and retina [9,20]. Because brain tissue is unable to make n-3 PUFAs, it is remarkably sensitive to adequate diet supplementation during all stages of CNS development – from embryonic differentiation to adulthood and aging [6,20,22,43,51]. Neural trauma and neurodegeneration are associated with significant disturbances in neuronal membrane phospholipid metabolism [13,28,36], suggesting that supplementation with n-3 PUFAs may be beneficial for recovery.

Omega-3 deficiency induces structural and functional abnormalities in the hippocampus, hypothalamus and cortex – brain areas that mediate spatial and serial learning [51]. Omega-3 deficiency significantly reduces the level of cerebral catecholamines, brain glucose transport capacity and glucose utilization, cyclic AMP level, and the capacity for phospholipid synthesis. Such a deficiency also markedly affects neuronal membrane fluidity, activity of membrane-bound enzymes, receptors and ion channels (e.g., Na^+,K^+-ATPase), production of neurotransmitters and brain peptides, gene expression (such as peroxisome prolifirator-activated receptor α (PPAR-α), sterol regulatory binding protein-1 (SREBP-1), carbohydrate regulatory element binding protein/Max-like factor X heterodimer (CREBP/Max-like factor X) [21], intensity of inflammation and synaptic plasticity [1,7,11,51].

Conversely, diet supplementation with DHA modulates gene expression, neurotransmitter release, restores synaptic activity reduced by age or trauma, and improves memory and learning abilities [12,17,33,36,37,47,49,50]. Omega-3 PUFAs possess strong anti-inflammatory properties, mediated partially by DHA's inhibition of arachidonic acid catabolism and modulation of cytokine levels [7,11]. Omega-3 PUFAs have also been found to protect in some animal models of acute ischemic brain injury [31,31,34,41].

Within the cell, the mitochondrial membrane is a primary site for n-3 PUFA incorporation [17,19,38,40,48]. A growing body of evidence has established that mitochondria are a key component in the signaling pathway(s) underlying cell death [2,14,15,29,44,45,47]. To some extent, mitochondria serve to integrate different apoptosis-inducing stimuli (Ca^{2+}, proapoptotic Bcl-2 family proteins, reactive oxygen species, etc.) and direct them into a common downstream pathway [2,14,29,45]. Mitochondria are enlisted to initiate the downstream stages of cell death through mitochondria permeability transition (MPT)-dependent and independent mechanisms. The MPT is a multiprotein pore complex of as yet unidentified structure that is presumably localized at the contact sites between the inner and outer mitochondrial membranes. The MPT

begins as a permeabilization of the inner membrane, which prevents buildup of a mitochondrial membrane potential, and leads to loss of the ability to sequester calcium from the medium, progressive osmotic swelling, disruption of the outer membrane, loss of matrix and intermembrane proteins, and initiation of caspase-dependent and caspase-independent cell death pathways [2,29]. Mitochondrial damage, occurring via the MPT, has been identified as a critical event in stroke and stroke-related injuries, secondary injury following brain trauma (TBI), and chronic neurodegeneration [2,15,29,45,47].

In light of the aforementioned links between mitochondria and cell death, mitochondria and n-3 PUFAs, and n-3 PUFAs and neuronal function, it is noteworthy that recent evidence shows that omega-3 PUFAs can modulate processes that contribute to the secondary degeneration of the CNS [18,27,36,49,50]. Particularly, administration of n-3 PUFAs after spinal cord compression injury in rats significantly increased neuronal survival and improved locomotive performance for up to 6 weeks after injury. Furthermore, pre-injury diet supplementation with omega-3 PUFAs prevented some TBI-induced effects - a reduction in synaptic plasticity, impaired learning ability, oxidative damage. Recent data suggest that eight weeks of dietary supplementation with DHA prevents induction of MPT in cardiac mitochondria [23]. These data, coupled with the above background, suggests that diets enriched in n-3 PUFAs affect mitochondria in a way that makes them more resistant to the oxidant- and calcium-mediated injury associated with both acute neurological injury and induction of the MPT.

The goal of present study was, therefore, to test directly the involvement of the MPT pathway in n-3 PUFA-mediated neuroprotection. Specifically, we determined whether 4 weeks dietary supplementation in rats with 1, 3 or 10% fish oil (FO) containing essential n-3 PUFAs, e.g., EPA and DHA, changes the resistance of isolated nonsynaptosomal brain and liver mitochondria to proapoptotic signals such as Ca^{2+} and prooxidants.

b. Explain how and why the animal species and model being used can address the scientific objectives and, where appropriate, the study's relevance to human biology.

The centerpiece of the study was the evaluation of dietary ώ-3 PUFA mediated effect on mitochondrial function isolated from brain and liver tissues. We have utilized animals, in a contrast, cell cultures, for instance, because they are the only model that can test the overall effects of diet on the organism including tissue specificity. Rats were chosen as they are well-characterized animal models of both diet and mitochondrial function, and they are the phylogenetically the lowest animal species that provided sufficient material to complete the planned experiments.

INTRODUCTION: Objectives

4 Clearly describe the primary and any secondary objectives of the study, or specific hypotheses being tested.

The objective of the study was to test a specific hypothesis - whether diets enriched in n-3 PUFAs affect mitochondria in a way that makes them more resistant to oxidant- and calcium-mediated injury.

METHODS: Ethical statement

5 Indicate the nature of the ethical review permissions, relevant licenses (e.g. Animal [Scientific Procedures] Act 1986), and national or institutional guidelines for the care and use of animals, that cover the research.

IACUC approval at BWH, following The Guide for the Care and Use of Laboratory Animals

METHODS: Study design

6 For each experiment, give brief details of the study design including:

a. The number of experimental and control groups.

There are 4 groups of 24 animals each. There is one control group fed with control (w/o fish oil) diet, and the three experimental groups each fed with 1, 3 or 10% fish oil diets.

b. Any steps taken to minimize the effects of subjective bias when allocating animals to treatment (e.g. randomization procedure) and when assessing results (e.g. if done, describe who was blinded and when).

We studied four diets simultaneously. The animals were ordered from Harlan to be approximately 3 weeks of age (20-25 days) and of similar body weight. The animals were assigned to cages randomly by the husbandry staff at unpacking and then the diets were assigned to each cage (2 animals per cage) randomly.

c. The experimental unit (e.g. a single animal, group or cage of animals). A time-line diagram or flow chart can be useful to illustrate how complex study designs were carried out.

The experimental unit for isolation of brain mitochondria is four animals (or, more specifically, brain tissues extracted from four animals were combined to isolate brain mitochondria). The experimental unit for isolation of liver mitochondria is one animal. Animals were always done by dietary group, e.g., four animals from the diet currently used.

EXPERIMENTAL

7 For each experiment and each experimental group, including controls, provide precise details of all procedures carried out. For example:

a. How (e.g. drug formulation and dose, site and route of administration, anesthesia and analgesia used [including monitoring], surgical procedure, method of euthanasia). Provide details of any specialist equipment used, including supplier(s).

No drugs and no specialist equipment. Complete dietary information is provided in this appendix.

.

b. When (e.g. time of day).

Sacrifice was at ~ 9 am, EST.

c. Where (e.g. home cage, laboratory, water maze).

Animal colony procedure room

d. Why (e.g. rationale for choice of specific anesthetic, route of administration, drug dose used).

NA

Sacrifice by decapitation without anesthesia following IACUC approved exemption due to known interference with assays.

EXPERIMENTAL: Experimental animals

8 a. Provide details of the animals used, including species, strain, sex, developmental stage (e.g. mean or median age plus age range) and weight (e.g. mean or median weight plus weight range).

We used FBNF1 male rats. Animals were brought into the colony at approximately 3 weeks of age and then maintained for 5 weeks on their assigned diet prior to sacrifice. The mean weight growth chart is presented below as Mean ± SD.

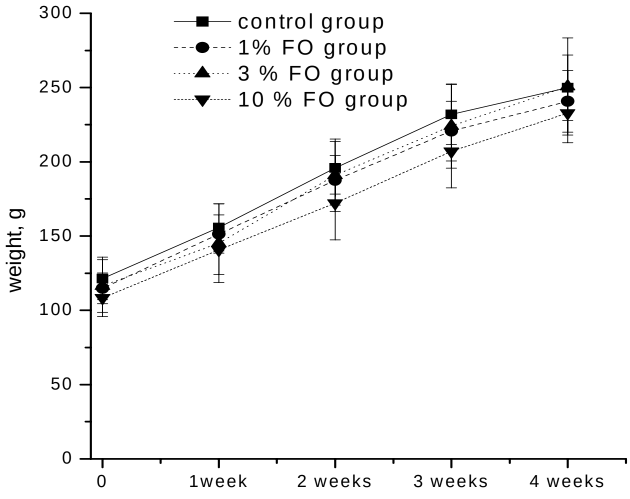

b. Provide further relevant information such as the source of animals, international strain nomenclature, genetic modification status (e.g. knock-out or transgenic), genotype, health/immune status, drug or test naïve, previous procedures, etc.

The animals were FBNF1 male rats acquired from Harlan Laboratories at approximately 3 weeks of age and of similar body weight. The diets were purchased from Research Diets, Inc. The animals were maintained in a non-barrier animal facility operated by Harvard Medical School.

EXPERIMENTAL: Housing and husbandry

9 Provide details of:

a. Housing (type of facility e.g. specific pathogen free [SPF]; type of cage or housing; bedding material; number of cage companions;

The animal facility is a non-barrier animal facility operated by Harvard Medical School. The animals were housed 2 per cage. The cages are standard plastic rat cages with wire lids (to hold food and water) and plastic, filtered tops. The animals live on the solid cage bottom with wood chip bedding (alpha chip). Cages are changed at least twice per week.

b. Husbandry conditions (e.g. breeding programme, light/dark cycle, temperature, quality of water etc for fish, type of food, access to food and water, environmental enrichment).

The light/dark cycle is 12 hour/12 hour. The temperature and humidity are maintained at 69 degrees and 45% respectively. The animals have ad lib access to food and water.

c. Welfare-related assessments and interventions that were carried out prior to, during, or after the experiment.

All work with animals must be approved by the IACUC committee at Brigham and Women's Hospital. Harvard Medical School has a full time staff of veterinarians and veterinary technicians that visit the facility on a regular weekly schedule. A veterinarian is on call during evenings, weekends, and holidays. The husbandry crew is present in the facility every day including weekends and holidays.

The animals on this study were not expected to suffer any ill effects clinically. And as expected, no animal appeared to be anything less than healthy at any time.

EXPERIMENTAL: Sample size

10 a. Specify the total number of animals used in each experiment, and the number of animals in each experimental group.

There are 4 groups of 24 animals each. Four animals from one dietary group were sacrificed at a time to obtain a sufficient yield of non-synaptosomal brain mitochondria. The liver tissue was extracted from only one animal (from the one of four animals used for brain extraction). The control group of animals was fed with control (w/o fish oil) diet and the three experimental animal groups were fed with one of either 1, 3 or 10% fish oil diet.

b. Explain how the number of animals was arrived at. Provide details of any sample size calculation used.

From experience it was estimated that this N (N=6) was sufficient to determine a trend of the dietary-mediated effect and conclude whether it is worth to continue the experiments in this direction.

c. Indicate the number of independent replications of each experiment, if relevant.

The experiments were replicated 6 times.

EXPERIMENTAL: Allocating animals to experimental groups

11 a. Give full details of how animals were allocated to experimental groups, including randomization or matching if done.

The diets were chosen in advance of the animals arriving. The animals were randomly assigned to cages, 2 per cage, by the husbandry crew at arrival. The cages were randomly assigned a diet.

b. Describe the order in which the animals in the different experimental groups were treated and assessed.

The experimental unit is one animal (or, more specifically, liver mitochondria isolated from one animal) and four animals for non-synaptosomal brain mitochondria isolated from four animals. Four animals from one dietary group were used for each experiment.

EXPERIMENTAL OUTCOMES

12 Clearly define the primary and secondary experimental outcomes assessed (e.g. cell death, molecular markers, behavioural changes).

The study design was focused to link dietary fish oil to changes in mitochondrial sensitivity to proapoptotic agents (e.g., calcium and prooxidants). It also tested the incorporation of n-3 polyunsaturated fatty acids in mitochondrial membrane to link the changes in membrane composition to function.

STATISTICAL METHODS

13 a. Provide details of the statistical methods used for each analysis.

Group comparisons of the effects of diet modulation on mitochondrial parameters were determined by a two-sample *t*-test (Origin 8.0). The significance of data was considered at *p*-value 0.05.

b. Specify the unit of analysis for each dataset (e.g. single animal, group of animals, single neuron).

Mitochondria isolated from single animal for the assessment of liver function and mitochondria isolated from four animals for the assessment of brain function were grouped by type of the diet.

c. Describe any methods used to assess whether the data met the assumptions of the statistical approach.

Group comparisons of the effects of diet modulation on mitochondrial parameters were determined by a two-sample *t*-test.

RESULTS: Baseline data

14 For each experimental group, report relevant characteristics and health status of animals (e.g. weight, microbiological status, and drug or test naïve) prior to treatment or testing. (This information can often be tabulated).

All animals were considered healthy at the time of sacrifice by a laboratory veterinarian (CLP).

NUMBERS ANALYZED

15 a. Report the number of animals in each group included in each analysis. Report absolute numbers (e.g. 10/20, not 50%) (Schulz et al., 2010).

The study evaluated 24 animals per dietary group. Brain tissue was dissected from 96 animals (e.g., 24 animals from each of the four dietary groups) for the brain mitochondria study; and liver tissue was dissected from 24 animals (e.g., from 6 animals from each of the four dietary groups) for liver mitochondria study.

b. If any animals or data were not included in the analysis, explain why.

Outcomes and estimation

Brain mitochondrial membrane n-3 PUFA incorporation was assessed in n=4 due to technical issues with mitochondrial preparations and/or assay.

16 Report the results for each analysis carried out, with a measure of precision (e.g. standard error or confidence interval).

Data were presented as the mean ± SEM.

Adverse events

17 a. Give details of all important adverse events in each experimental group.

None

b. Describe any modifications to the experimental protocols made to reduce adverse events.

NA

DISCUSSION: Interpretation/scientific implications

18 a. Interpret the results, taking into account the study objectives and hypotheses, current theory and other relevant studies in the literature.

Obtained results rejected the initial hypothesis.

b. Comment on the study limitations including any potential sources of bias, any limitations of the animal model, and the imprecision associated with the results (Schulz et al., 2010).

The animal model is unusual in that the strain, FBNF1, is a hybrid. This strain is a healthy and hardy animal that is not prone to specific disease or obesity. These animals were also fed a low fat (10% total fat) diet.

Isolation of brain mitochondria from four rats at a time increases heterogeneity of mitochondrial properties and their response to endogenously added stimuli, such as calcium or prooxidants.

c. Describe any implications of your experimental methods or findings for the replacement, refinement or reduction (the 3Rs) of the use of animals in research.

None that are obvious.

DISCUSSION: Generalisability/translation

19 Comment on whether, and how, the findings of this study are likely to translate to other species or systems, including any relevance to human biology.

The present study tested and rejected the most direct and parsimonious mechanism linking dietary supplementation and brain and liver mitochondria functional parameters and injury mechanisms. The absence of a protective effect of diet in an *in vitro* model of ischemic injury (Ca^{2+} overloading), despite the observed changes in plasma and mitochondrial phospholipid content, suggests that increased resistance to Ca^{2+}- and oxidant-

mediated mitochondrial damage is not central to the well-documented neuroprotection induced by omega-3 PUFAs.

FUNDING

20 List all funding sources (including grant number) and the role of the funder(s) in the study.

The study reported was funded by HCNRC pilot feasibility project (IGS, PI), P30-DK040561, NIH (W. Allan Walker, PI), U01-ES16048 (BSK, PI), a part of the NIH Genes and Environment Initiative (GEI), and discretionary funds from Brigham and Women's Hospital (to BSK).

ARRIVE CRITERIA Literature

1: Kilkenny C, Browne WJ, Cuthill IC, Emerson M, Altman DG. Improving bioscience research reporting: The ARRIVE guidelines for reporting animal research. J Pharmacol Pharmacother. 2010 Jul;1(2):94-9. PubMed PMID: 21350617; PubMed Central PMCID: PMC3043335.

2: Kilkenny C, Browne W, Cuthill IC, Emerson M, Altman DG; National Centre for the Replacement, Refinement and Reduction of Amimals in Research. Animal research: reporting in vivo experiments--the ARRIVE guidelines. J Cereb Blood Flow Metab. 2011 Apr;31(4):991-3. Epub 2011 Jan 5. PubMed PMID: 21206507; PubMed Central PMCID: PMC3070981.

3: Kilkenny C, Altman DG. Improving bioscience research reporting: ARRIVE-ing at a solution. Lab Anim. 2010 Oct;44(4):377-8. Epub 2010 Jul 21. PubMed PMID: 20660161.

4: Kilkenny C, Browne W, Cuthill IC, Emerson M, Altman DG; NC3Rs Reporting Guidelines Working Group. Animal research: reporting in vivo experiments: the ARRIVE guidelines. Br J Pharmacol. 2010 Aug;160(7):1577-9. PubMed PMID: 20649561; PubMed Central PMCID: PMC2936830.

5: McGrath JC, Drummond GB, McLachlan EM, Kilkenny C, Wainwright CL. Guidelines for reporting experiments involving animals: the ARRIVE guidelines. Br J Pharmacol. 2010 Aug;160(7):1573-6. PubMed PMID: 20649560; PubMed Central PMCID: PMC2936829.

6: Kilkenny C, Browne WJ, Cuthill IC, Emerson M, Altman DG. Improving bioscience research reporting: the ARRIVE guidelines for reporting animal research. PLoS Biol. 2010 Jun 29;8(6):e1000412. PubMed PMID: 20613859; PubMed Central PMCID: PMC2893951.

7: Kilkenny C, Browne W, Cuthill IC, Emerson M, Altman DG; NC3Rs Reporting Guidelines Working Group. Animal research: reporting in vivo experiments: the ARRIVE guidelines. J Gene Med. 2010 Jul;12(7):561-3. PubMed PMID: 20607692.
